# Circulating Polyploid Giant Cancer Cells, a Potential Prognostic Marker in Patients with Carcinoma

**DOI:** 10.3390/ijms25189841

**Published:** 2024-09-11

**Authors:** Ludmilla Thomé Domingos Chinen, Jacqueline Aparecida Torres, Vinicius Fernando Calsavara, Angelo Borsarelli Carvalho Brito, Virgílio Sousa e Silva, Roberto Gabriel Santiago Novello, Thaissa Carvalho Fernandes, Alessandra Decina, Roger Dachez, Patrizia Paterlini-Brechot

**Affiliations:** 1 Hcor Research Institute, São Paulo 04004-030, Brazil; ltdchinen@gmail.com; 2 Hospital Amaral Carvalho, Jaú 17210-080, Brazil; 3 Department of Farmacology, Federal University of São Paulo (UNIFESP), São Paulo 04044-020, Brazil; jacque_a_torres@hotmail.com; 4 Department of Computational Biomedicine, Biostatistics Shared Resource, Cedars-Sinai Cancer Center, Los Angeles, CA 90069, USA; vinicius.calsavara@cshs.org.com; 5 Department of Clinical Oncology, A.C. Camargo Cancer Center, São Paulo 01509-900, Brazil; 6 Rarecells Faculté de Médecine Necker, 160 Rue de Vaugirard, 75015 Paris, France; alessandra.decina@rarecells.com; 7 Cytopathology Laboratory Innodiag, F-92100 Boulogne-Billancourt, France; roger.dachez@gmail.com

**Keywords:** polyploid giant cancer cells, cancer giant cells, prognostic marker, liquid biopsy, lung cancer, colon cancer, gastric cancer

## Abstract

Polyploid Giant Cancer Cells (PGCCs) have been recognized as tumor cells that are resistant to anticancer therapies. However, it remains unclear whether their presence in the bloodstream can be consistently detected and utilized as a clinical marker to guide therapeutic anticancer regimens. To address these questions, we conducted a retrospective study involving 228 patients diagnosed with six different types of carcinomas (colon, gastric, NSCLC, breast, anal canal, kidney), with the majority of them (70%) being non-metastatic. Employing a highly sensitive liquid biopsy approach, ISET^®^, and cytopathological readout, we isolated and detected circulating PGCCs in the patients’ blood samples. PGCCs were identified in 46 (20.18%) out of 228 patients, including in 14.47% of 152 non-metastatic and 29.85% of 67 metastatic cases. Patients were subsequently monitored for a mean follow up period of 44.74 months (95%CI: 33.39–55.79 months). Remarkably, the presence of circulating PGCCs emerged as a statistically significant indicator of poor overall survival. Our findings suggest that circulating PGCCs hold promise as a reliable prognostic indicator. They underscore the importance of further extensive investigations into the role of circulating PGCCs as a prognostic marker and the development of anti-PGCC therapeutic strategies to improve cancer management and patient survival.

## 1. Introduction

Despite significant financial investments in anticancer therapies, the mortality rate among cancer patients continues to be unacceptably high, with nearly 10 million deaths occurring annually worldwide [1]. This persistent challenge highlights our incomplete understanding of the fundamental mechanisms underlying cancer growth and recurrence, revealing the limitations of current anticancer modalities in effectively eradicating the disease.

Recently, a groundbreaking perspective on cancer development and recurrence has emerged, drawing from profound insights into tumor pathology and the origins of life [2]. This paradigm proposes that somatic cells, when subjected to stress, can undergo a process of dedifferentiation akin to a blastomere-like pathway, giving rise to Polyploid Giant Cancer Cells (PGCCs). While PGCCs have been observed in cancer tissues for decades, they were previously believed to be non-dividing cells. However, recent data reveal that PGCCs can indeed undergo division through endoreduplication, a process reminiscent of cleavage divisions seen in blastomeres, which may lead to dedifferentiation of somatic cells. At the core of this paradigm shift is the notion that tumors originate from a stem cell whose differentiation has become uncoupled from its proliferation program. Consequently, this leads to a halt in stem cell maturation, with the degree of malignancy determined by the stage at which this maturation arrest occurs.

This perspective underscores the pivotal role of PGCCs in the genesis of cancer, shedding new light on our understanding of the disease’s underlying mechanisms. It is also widely recognized that the tumor environment can experience drastic changes due to factors such as diminished blood supply or the effects of anticancer therapies. To thrive amidst these challenges, cancer cells must adapt quickly, enabling them to survive in diverse and hostile conditions. Recent research has shed light on the role of PGCCs, also known as polyaneuploid cancer cells (PACCs), in the context of cancer resistance and survival. Studies, like the one conducted by Fei et al. and by Mallin et al. [3,4], highlight the remarkable ability of PGCCs to withstand anticancer treatments and exhibit heightened motility and invasion capabilities. These traits are pivotal in processes like metastasis formation, cancer recurrence, and ultimately, in determining the lethality of the disease.

The purpose of this study was (1) to retrospectively investigate whether the presence of PGCCs in the bloodstream of cancer patients can be consistently detected by using a highly sensitive approach to isolate intact tumor cells from blood, ISET^®,^ and cytopathological readout, and (2) if PGCCs can be used as a clinical prognostic marker to guide anticancer regimens. We thus conducted a retrospective analysis of blood samples from patients diagnosed with various types of carcinomas and previously analyzed for the presence of CTCs. Patients were followed up for a mean period of 44.74 months. We took advantage of ISET^®^, which allowed the establishment of a biobank of CTCs, to analyze the PGCC presence and morphology and correlate these findings with patients’ clinical outcomes.

While our findings are preliminary, they reveal a statistically significant association, in all patients studied together and in patients with colon cancer, our largest group, between the presence of circulating PGCCs and poor overall survival. This suggests that the cytological detection of circulating PGCCs may serve as a valuable prognostic indicator for assessing disease progression and patient outcomes.

## 2. Results

We conducted a comprehensive retrospective analysis of blood samples obtained from a total of 228 patients with various types of carcinomas, including colon, non-small cell lung cancer (NSCLC), gastric, breast, anal canal, and kidney cancers, previously enrolled in distinct prospective trials at the A. C. Camargo Cancer Center. Patients were followed up for a mean period of 44.74 months. The impact of their clinical-pathological features on OS and PFS is reported in the Supplementary Materials (Figures S1–S9).

We used the highly sensitive ISET^®^ technology to isolate CTCs and Polyploid Giant Cancer Cells (PGCCs) from blood and carefully stored both stained and non-stained ISET^®^ membranes and relative spots obtained from patients enrolled in previously conducted clinical studies, building an ISET^®^ CTC biobank and ensuring the opportunity for further examination and research. For the present study, we conducted a retrospective analysis of the ISET^®^ spots, specifically targeting the identification of PGCCs.

**Figure 1:**
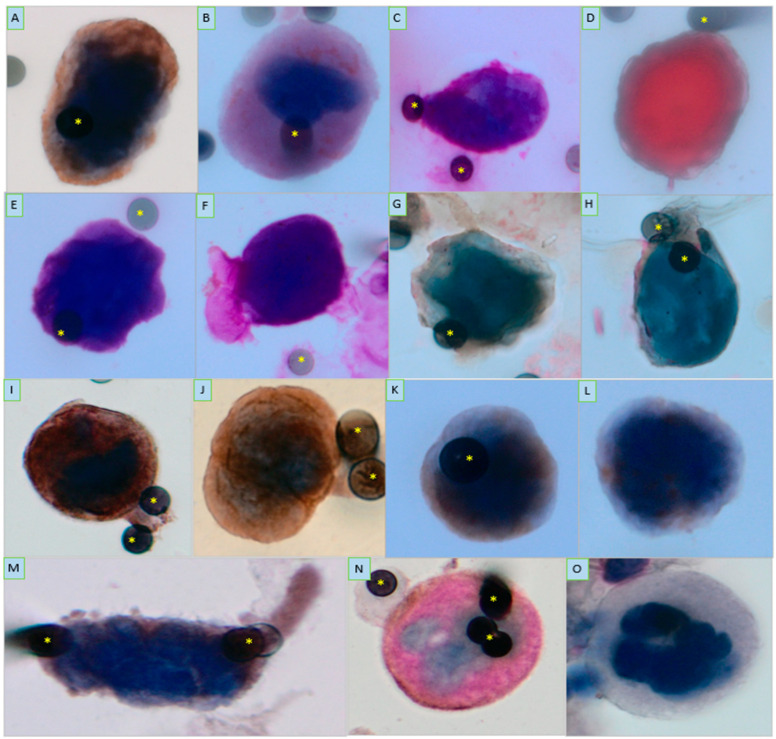
Panel of pictures of Polyploid Giant Cancer Cells (PGCCs) from different types of carcinoma. (**A**) PGCCs from non-metastatic breast cancer patient stained with HER2 antibody revealed with 3,3′-Diaminobenzidine (DAB); (**B**) PGCCs of non-metastatic canal anal cancer, expressing PD-L1 and RAD23B antibodies revealed with DAB and Magenta chromogens, respectively; (**C**) PGCCs of non-metastatic canal anal cancer, stained with ERCC1 revealed with Magenta chromogen; (**D**) PGCCs derived from non-metastatic colon cancer stained with anti-TIMP and anti-MMP-2 revealed with DAB and Magenta chromogens, respectively; (**E**,**F**) Metastatic renal cancer PGCCs expressing BAP1 and NCAD, with the magenta chromogen used for both revelations; (**G**,**H**) PGCCs found in non-metastatic gastric cancer, expressing HER2 revealed with DAB chromogen; (**I**–**K**) PGCCs of metastatic NSCLC stained with TGFβRI and revealed with DAB chromogen; (**L**) PGCCs of metastatic NSCLC stained with EGFR and revealed with DAB chromogen; (**M**) PGCCs derived from metastatic NSCLC expressing CD47 revealed with DAB chromogen; (**N**) PGCC derived from metastatic NSCLC expressing TGFβRI and CD45 and revealed with DAB and magenta chromogens, respectively; (**O**) PGCCs of metastatic NSCLC without any staining, revealed by H&E. All images were taken at 400× magnification using a light microscope (Research System Microscope BX61-Olympus, Tokyo, Japan) coupled to a digital camera (SC100-Olympus, Tokyo, Japan). Morphological characteristics of PGCC are observed: cells with diameter more than 40 µm, high nuclei–cytoplasm ratio, mononucleated or multinucleated cells. The micropores, which are nominal 8 μm in diameter, of ISET^®^ membrane are identified by yellow asterisks.

Our analysis encompassed 1094 spots derived from 228 patients diagnosed with different types of carcinomas. We conducted the examination of a mean number of 4.89 spots per patient (SD 1.13; range: 1–10), with a median 3.0 spots per patient, which is a reliable number of spots as previously reported [5]. Although we stained the spots with several immunostainings according to the cancer type (Figure 1), the PGCC analysis here only considers the presence/absence of PGCCs based on the cytomorphological characteristics described in the Methods section. However, the most commonly expressed markers in PGCCs were as follows (number of positive PGCC/tested PGCC): CD47 (7/46 PGCCs; 15.21%); ERCC1 (6/46; 13.04%); HER-2 (6/46; 13.04%); CD45 (5/46; 10.86%); TGFβRI (4/46; 8.69%); and HIF (4/46; 8.69%). Other markers that we evaluated or co-evaluated are as follows: vimentin (3/46; 6.52%), β-galactosidase (3/46; 6.52%), TYMS (3/46; 6.52%), EGFR (2/26; 4.35%), BAP-1(2/26; 4.35%), PD-L1 (2/26; 4.35%), CD133 (2/26; 4.35%) and ESR (1/46; 2.17%), MC1R (1/46; 2.17%), MMP-2 (1/46; 2.17%), RAD-23B (1/46; 2.17%), SETD2 (1/46; 2.17%), and TIMP1 (1/46; 2.17%). From these data, we can observe that the PGCC cell population is extremely heterogeneous. Dedicated studies are thus needed to further explore the immune molecular profile of circulating PGCCs in different cancer types.

We observed an average of 0.44 PGCC per patient (SD: 1.23; range: 0–9 PGCC) and an average of 0.15 PGCC/mL blood (SD: 0.41; range: 0–3.00 PGCC/mL) when considering the 228 patients, with and without PGCCs. CTCs were present in 203 out of 228 patients, with a median count of 2.50 CTCs/mL (range: 0–51 CTCs/mL). Notably, within this cohort, we noted an average of 0.13 PGCC per CTC (SD: 0.71; range: 0–9.09 PGCC per CTC).

Considering only patients with PGCCs, we observed a mean of 2.17 PGCC per patient (SD: 1.96; range: 1–9 PGCC) and an average of 0.72 PGCC/mL (SD: 0.65; range: 0.25–3.00 PGCC). In addition, the mean was 0.62 PGCC per CTC (SD: 1.48; range: 0.03–9.09 PGCC per CTC).

### 2.1. PGCCs in All Patients Studied

#### 2.1.1. PGCC Presence, Clinical-Pathological Features and Follow Up

We evaluated the potential clinical impact of the presence of PGCCs at diagnosis or at the time when metastases were detected, before any subsequent therapy (see Methods). PGCCs were detected in 46 patients out of 228 (20.18%), including 29/125 (23.20%) female and 17/101 (16.83%) male of 226 patients with informed gender (Table 1). We studied 67 (30.59%) patients with metastatic disease and 152 (69.41%) with non-metastatic disease among the 219 patients with informed metastatic/non-metastatic status. PGCCs were statistically significant more frequently found in patients with metastatic disease (n = 20/67) in comparison with non-metastatic ones (n = 22/152) (29.85% versus 14.47%; *p* = 0.008) and in patients with high mean and median CTC/mL (*p* = 0.007).

#### 2.1.2. PGCC Presence and Overall Survival

**Figure 2:**
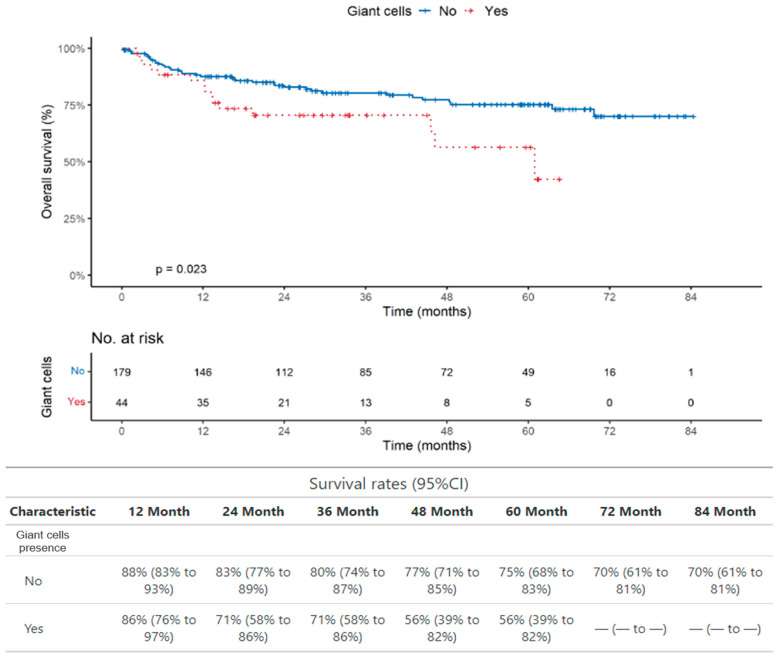
Overall survival analysis in the whole group of patients. The PGCC presence is significantly associated with poor OS.

We observed, at 5 years, 75% OS in patients without PGCCs (95%CI: 68% to 83%) versus 56% (95%CI: 39% to 82%) in patients with PGCCs. The univariable Cox regression analyses showed that the presence of PGCCs is statistically significantly associated with poor overall survival (OS) (HR = 1.990, 95%CI: 1.087 to 3.644; *p* = 0.023) when all types of patients were evaluated together (Table 2 and Figure 2). We did not find any association of PGCC/mL with OS and PFS.

#### 2.1.3. PGCC Presence and Progression-Free Survival

The analysis of progression-free survival (PFS) did not demonstrate a statistically significant association between PGCCs and PFS.

#### 2.1.4. CTC Presence and Overall Survival

**Figure 3:**
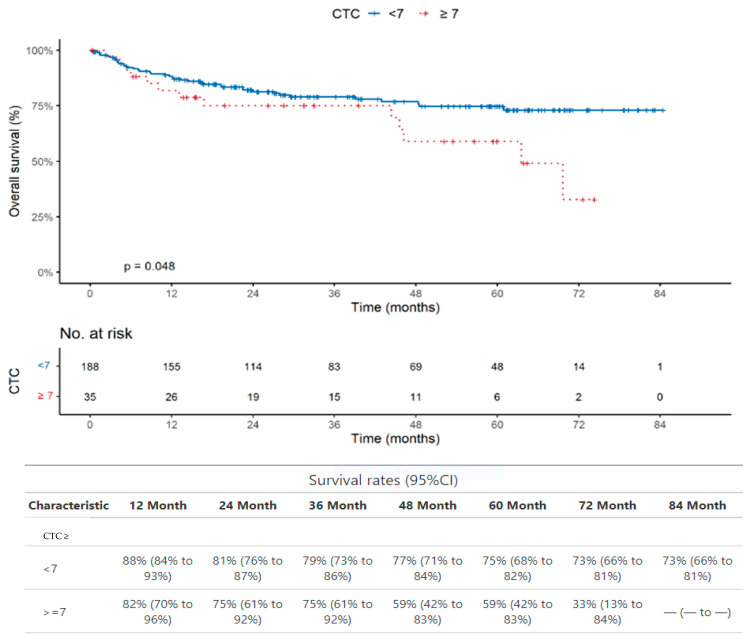
Overall survival analysis in the whole group of patients. The CTC presence with cut-off of 7 is significantly associated with poor OS (*p* = 0.048).

The CTC presence is not significantly associated with poor OS (*p* = 0.84). However, the same analysis performed with a cut-off of seven CTCs (Figure 3) showed that the CTC presence with cut-off of seven is significantly associated with poor OS, although at a border line level (*p* = 0.048).

### 2.2. PGCC Analysis in Patients with Specific Tumor Types

Among the different types of cancers studied, colon cancer prevailed as the most frequently evaluated, accounting for 76 cases (33.33%), followed by gastric cancer with 51 cases (22.37%), and NSCLC with 45 cases (19.74%). PGCC and CTC data per each tumor type are provided in Table 3.

Colon cancer: For colon cancer, seventy-six patients were included, including thirty-seven (50%) females, thirty-seven (50%) males, and two patients with no gender specified. PGCCs were observed in 6/37 (16.22%) female patients and in 3/37 (8.11%) male patients, without any association between gender and PGCCs. We observed a mean of 0.26 PGCC (SD: 1.12; range: 0–9.00 PGCC), a mean of 0.08 PGCC/mL of blood (SD: 0.29; range: 0–2.25 PGCC/mL), a mean of 0.03 PGCC/CTC (SD: 0.13; range: 0–0.97 PGCC/CTC) (Table 3), and a mean of 3.84 CTC/mL (SD: 4.09; range: 0–24.25 CTC/mL).

**Figure 4:**
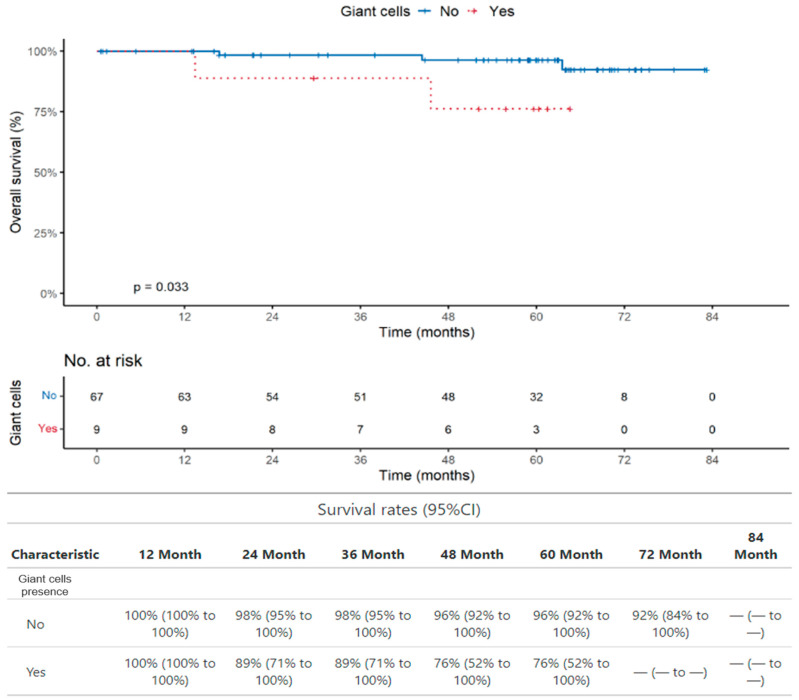
Overall survival. Association between PGCCs and poor OS in patients with colon cancer (*p* = 0.033).

We found a statistically significant association, at 5 years of PGCCs with poor OS (*p* = 0.033), with an OS of 96% in the absence of PGCCs (95% CI: 92% to 100%) versus an OS of 76% with the presence of PGCCs (95% CI: 52% to 100%) (Figure 4). The analysis of PFS did not demonstrate any statistically significant association with PGCCs.

**Figure 5:**
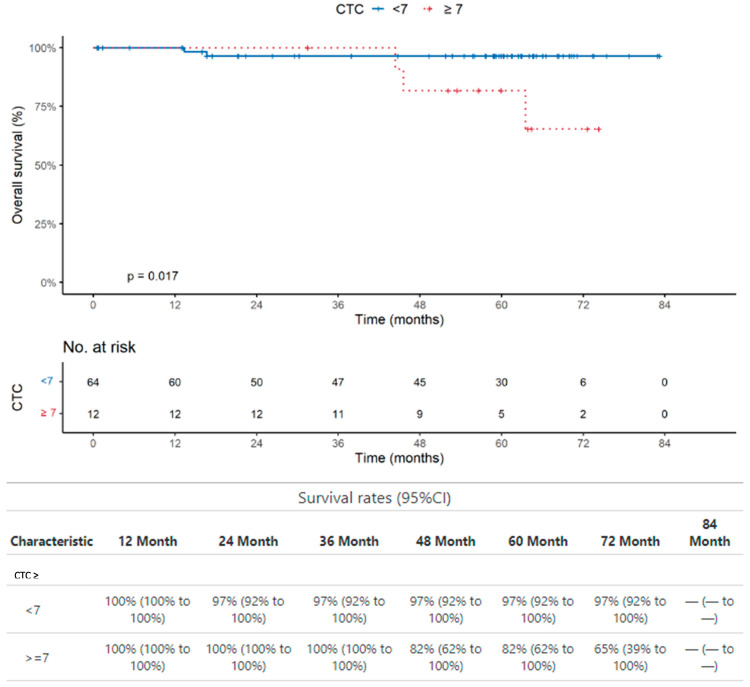
Overall survival analysis in patients with colon cancer. The CTC presence with cut-off of 7 is significantly associated with poor OS (*p* = 0.017).

The CTC presence in patients with colon cancer is not significantly associated with poor OS (*p* = 0.83). However, the same analysis performed with a cut-off of seven CTCs (Figure 5) showed that the CTC presence with cut-off of seven is significantly associated with poor OS (*p* = 0.017).

Gastric Cancer: We included 51 patients with gastric cancer, including 20 (39.22%) females and 31 (60.78%) male. PGCCs were observed in 6/20 (30%) female patients and in 3/31 (9.68%) male patients. There was no association between gender and PGCCs in gastric cancer. PGCCs were not found in patients with a positive lymph node, as only two patients out of thirty-two with positive lymph nodes showed PGCCs (6.25%; *p* = 0.046). We observed a mean of 0.33 PGCC (SD: 0.84; range: 0–4.00 PGCC), a mean of 0.11 PGCC/mL of blood (SD: 0.27; range: 0–1.00 PGCC/mL), and a mean of 0.10 PGCC/CTC (SD: 0.59; range: 0–4.17 PGCC/CTC) (Table 3). We also noted a mean of 5.45 CTC/mL (SD: 9.14; range: 0–51.00 CTC/mL). The analysis of PFS did not demonstrate any statistically significant association with PGCCs.

Kidney Cancer: We included twelve patients with kidney cancer, including three (25.00%) females and nine (75.00%) males. PGCCs were observed in 2/9 (22.22%) male patients and were not observed in female patients. There was no association between gender and PGCCs in kidney cancer patients. We observed a mean number of 0.83 PGCC in our patients (SD: 2.33; range: 0–8.00 PGCC), a mean of 0.21 PGCC/mL of blood (SD: 0.58; range: 0–2.00 PGCC/mL), and a mean of 0.26 PGCC/CTC (SD: 0.63; range: 0–2.00 PGCC/CTC) (Table 3). We also observed a mean of 2.46 CTC/mL (SD: 2.43; range: 0.25–7.75 CTC/mL). We did not observe any statistically significant association between PGCCs and different clinical features in patients with kidney cancer.

Non-Small Cell Lung Cancer (NSCLC): We included 45 patients with NSCLC, including 25 (55.56%) females and 20 (44.44%) males. PGCCs were observed in 10/25 (40.00%) female patients and in 6/20 (30.00%) male patients, without any association between gender and PGCCs. We found a mean number of 0.73 PGCC in our patients (SD: 1.56; range: 0–8.00 PGCC), with a mean of 0.24 PGCC/mL of blood (SD: 0.52; range: 0–2.67 PGCC/mL), and mean of 0.07 PGCC/CTC (SD: 0.17; range: 0–1.01 PGCC/CTC) (Table 3).

In patients with NSCLC, the presence of PGCCs was strongly and significantly associated with the presence and number of CTC/mL (*p* < 0.001), with a mean of 3.20 CTC/mL (SD: 3.42; range: 0–11.33 CTC/mL) when PGCCs were present. We did not observe any statistical association of PGCC presence and clinical features in patients with NSCLC.

Anal Canal Cancer: We included fifteen patients with anal canal cancer, eleven (73.33%) females and four (26.67%) males. PGCC were observed in 3/11 (27.27%) female patients and in 3/4 (75.00%) male patients, with no association found between gender and PGCCs. We observed a mean number of 0.93 PGCC (SD: 1.39; range: 0–4.00 PGCC), with a mean of 0.44 PGCC/mL of blood (SD: 0.82; range: 0–3.00 PGCC/mL) and mean of 0.94 PGCC/CTC (SD: 2.35; range: 0–9.09 PGCC/CTC) (Table 3). A mean of 1.10 CTC/mL (SD: 1.11; range: 0.33–3.66 CTC/mL) was noted. We found no statistically significant association of clinical features and PGCCs in patients with anal canal cancer.

Breast Cancer: We included 29 women with breast cancer. PGCCs were observed in 4/29 patients (13.79%). We found a mean number of 0.21 PGCC (SD: 0.56; range: 0–2.00 PGCC), with a mean of 0.06 PGCC/mL of blood (SD: 0.17; range: 0–0.67 PGCC/mL), a mean of 0.03 PGCC/CTC (SD: 0.12; range: 0–0.67 PGCC/CTC) (Table 3), and also a mean of 4.74 CTC/mL (SD: 6.97; range: 0–30.50 CTC/mL). No statistically significant association was observed between PGCCs and clinical features in patients with breast cancer.

## 3. Discussion

We have retrospectively studied the presence of circulating Giant Cells having the characteristics of Polyploid Giant Cancer Cells (PGCC) in 228 patients with different types of cancer (colon, gastric, NSCLC, breast, and anal carcinomas). We have identified PGCCs by cytomorphological analysis as cells with a size larger than 40 microns and cancer-like cytomorphological aspects (see Methods).

Several other studies have been focused on Giant Cells, defined with different names, including cancer-associated macrophage-like cells (CAMLs) [6], neoplastic-immune hybrid cells [7], Polyploid Giant Cancer Cells (PGCC) [8,9], polyaneuploid cells (PACCs) [10], blastomere-like cancer cells [11], osteoclast-like cancer cells [12], circulating giant tumor–macrophages fusion cells [13], hybrid cells [14], and pleomorphic cancer cells [15]. It is unclear if different names correspond to different cell characteristics or not. However, the majority of reports have been conducted using immunofluorescence approaches, which do not allow a precise study of the cellular and, in particular, nuclear characteristics. Giant Cells are known to be associated with chronic infections and inflammations [16] and can circulate in patients without cancer. Furthermore, cellular markers which are diagnostic for tumor nature are lacking. In this setting, we think that the possibility to assess cellular and nuclear characteristics of tumor nature through a careful cytomorphological analysis is important to distinguish true PGCCs from Giant Cells potentially related to chronic inflammation/infection. In the present study, like in very few other studies [17], the cytopathological examination is used to identify circulating PGCCs and to define their tumor-like morphological characteristics. By this approach, we have found that the presence of PGCCs is significantly increased in patients with metastatic versus non-metastatic cancers. In fact, PGCCs have been suggested to be the first cells triggering tumorigenesis and cancer recurrence [3]. Consistently, we find an increased number of patients with metastases having circulating PGCCs. PGCCs, or a subtype of them, are hybrid cells including a macrophage-like cell. In view of this finding, it is not surprising that PGCCs may circulate and be found in peripheral blood.

We also found that the presence of circulating PGCCs is significantly associated with poor overall survival (OS) when we consider the whole group of 228 patients (*p* = 0.023) and patients with colon cancer (76 patients; *p* = 0.033), which is the largest group in our cohort.

It is noteworthy that the presence of CTCs assessed in the same samples we used for the analysis of PGCCs did not show any association with poor overall survival (*p* = 0.84 and *p* = 0.46 for all patients studied and for patients with colon cancer, respectively). Interestingly, the use of a cut-off of seven CTCs showed a significant association between CTC > 7 and OS in all the 228 patients studied (*p* = 0.048), although borderline (Figure 2), and in the 76 patients with colon cancer (*p* = 0.017) (Figure 4).

The data we obtained are consistent with the view that CTCs are a heterogenous group of cells and that by introducing the cut-off, we minimize the impact of CTCs having low or no prognostic value. The presence of PGCCs, on the other hand, shows the “per se” prognostic value without the need of a cut-off, indicating the higher prognostic value of PGCCs as compared to CTCs.

Thus, circulating PGCCs, which are counted among CTCs since they have cancer features, can be considered a subgroup of CTCs with increased malignant potential.

It is tempting to speculate that patients with more malignant tumors generate more PGCCs after treatment and that some of them circulate in blood. Since PGCCs are known to be at the origin of recurrence, through the generation of highly proliferative cancer cells, the process will lead to uncontrolled tumor cells expansion and eventually to death.

Some studies, performed with immunofluorescence methods, have reported the importance of circulating Giant Cells as prognostic tumor markers [6,18]. We think that both approaches, immunofluorescence and cytomorphology/immunolabelling, are important and complementary. For instance, further cytological studies could identify subtypes of PGCCs with a single nucleus or multiple nuclei and specific markers, allowing the assessment of their clinical impact. In fact, the field of circulating Giant Cells needs more analyses to classify them and to explore the malignant potential of the different subtypes.

In this study, we have applied cytomorphology and immunolabelling to the study of circulating PGCCs. Although our immunolabelling results are limited and not suitable to draw conclusions, they show a high level of phenotypic heterogeneity of PGCCs and underscore the importance of combined cytological and immunolabelling analyses to characterize circulating GCs and distinguish inflammation-associated GCs from cancer-associated GCs [19].

In conclusion, we report here preliminary results on the cytomorphological detection of circulating Giant Cells, having a profile of PGCCs, in patients with different types of carcinomas. Although this study is retrospective and has limitations in the number of patients with different cancer subtypes and cancer stages, it reveals that the presence of circulating PGCCs is significantly associated with the presence of metastases and to a shorter OS. Further work, using immuno-cytomorphological analyses, has to be planned to study the presence, subtype, and clinical impact of PGCCs circulating in the blood of cancer patients.

## 4. Materials and Methods

**Figure 6:**
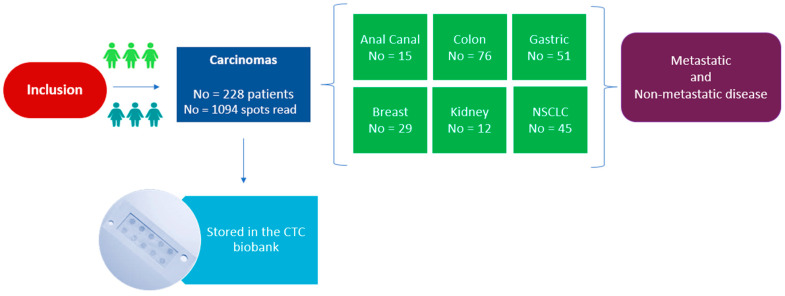
Patients’ inclusion. Flowchart showing the number of patients included per cancer type.

We retrospectively analyzed the blood of 228 patients with different types of carcinomas (76 colon cancers, 15 locally advanced canal anal cancers, 51 gastric cancers, 29 breast cancers, 12 kidney cancers, and 45 non-small cell lung cancers) treated at the A. C. Camargo Cancer Center (São Paulo, Brazil) between 2016 and 2023 (Table 1, Figure 6). For all patients, blood samples were collected at diagnosis or during follow up at the time when metastasis was detected, before any subsequent therapy (Table 1 and Table 4). Patients’ ECOG performance status was ranging from 0, 1 to 2; male-to-female ratio was 44.69%. Among the 228 patients, 152 had non-metastatic cancers and 67 had metastatic cancers. No information was obtained for the remaining 9 patients (Table 1 and Table 4). Patients had a mean follow up of 44.74 months (95%CI: 33.39–55.79; range: 0.33–86.12 months). The clinical-pathological features of patients are described in Table 1 and Table 4. The studies on the different cancer types were approved by institutional review boards (anal canal cancers: 2696/19; colon cancers: 1367/10 and 2141/15B; NSCLC: 2496/18C; gastric cancers: 2134/15; breast cancers: 2345/14 and 2861/20; kidney cancers: 2855/20).

### 4.1. PGCC Detection by ISET^®^ Technology

Our laboratory is highly specialized in circulating tumor cells (CTCs) and their detection and analysis in patients with different types of tumors [14,17,19,20,21,22,23,24,25,26]. CTCs are isolated from blood using the hypersensitive ISET^®^ technology, able to capture down to 1 CTC in 10 mL of blood [27]. Blood samples are collected on EDTA and filtered through a polycarbonate membrane, composed of 10 circular areas (spots) containing thousands of micropores that are nominal 8 μm in diameter. Each spot concentrates the CTCs present, before filtration, in one mL of blood. Tumor cells have a diameter larger than 8 μm [28]. The excellent sensitivity of the ISET^®^ technology is based on the combination of specific buffers, membrane, consumables, and device characteristics. Fixed CTCs are captured on the membrane without the need for antibodies, enabling their storage at −20 °C. We have built an ISET^®^-CTC biobank since 2011, where we preserve all membranes and spots, whether stained or unstained, at −20 °C.

For the present study, we cytologically evaluated 1094 spots stained with different antibodies, from 228 patients with different types of carcinomas. We thus analyzed a mean number of 4.89 spots per patient.

Using a light microscope (Research System Microscope BX61-Olympus, Tokyo, Japan) coupled with a digital camera (SC100-Olympus, Tokyo, Japan), we screened each spot searching for PGCCs. We considered cells stained with cytological staining and/or labeled with different types of antibodies. Cells having the following morphological characteristics were defined as PGCCs: cells larger than 40 microns and having cancer-like cytomorphological aspects, such as anisonucleosis, nuclear hyperchromatism, high nucleo-cytoplasmic ratio, atypical nucleoli, and atypical cell shape. Each detected Giant Cell was photographed and reassessed, to confirmation, by three experts in CTCs and a cytopathologist expert in CTCs and Giant Cells. Examples of PGCCs are shown in Figure 1.

### 4.2. Immunostaining of Spots by ISET^®^ Technology

For immunostaining of spots, we performed dual immunocytochemistry (ICC), combining each antibody of interest (HER2, PD-L1, RAD23B, ERCC1, anti-TIMP, anti-MMP-2, BAP1, NCAD, TGFβRI, EGFR, and CD47) for each project with anti-CD45 for leukocyte cell exclusion in the majority of the times. We put ISET^®^ membrane’s spots in a 24-well plate. In each well, to perform antigenic retrieval, 1 mL of Retrieval Solution (EnVision FLEX Dako^TM^, low pH, Dako^TM^, Santa Clara, CA, USA) was added and heated (three repetitions of 1:40 min heating in a microwave oven), followed by hydration with tris-buffered saline (TBS), dilution 1:10. The cells were permeabilized with TBS 0.2% + Triton X-100 for 5 min at room temperature and incubated with Endogenous Peroxidase Blocked (EnVision FLEX—Dako^TM^) for 5 min in the dark, for enzymatic blockade. After each step, the spots were washed once with TBS. The spots were incubated overnight with antibodies diluted in TBS 10% fetal serum, followed by incubation with Horseradish Peroxidase—HRP (EnVision FLEX—Dako^TM^)—for 20 min. The revelation was made using chromogen Diaminobenzidine 3.3′ (DAB) (Dako^TM^), incubated for 10 min. Before adding the second antibody, the cells were incubated with sulfuric acid (0.1 M) to prevent possible non-specific reactions of the HRP molecules with the second chromogen. After incubating the second antibody for two hours, we performed a new incubation with HRP for 20 min. The revelation was made using magenta chromogen (EnVision FLEX—Dako^TM^) incubated for 5 min (after each step, the spots were washed once with TBS), followed by hematoxylin staining for 2 min. Then, the spots were washed three times with distilled water. Mounting Medium—Dako was used to bond the spots in glass microscope slide.

In this study, our aim was to evaluate the clinical impact of the presence of PGCCs based on the cytological criteria described above. We report the immunostaining positivity (see Results) but do not draw any conclusion based on this parameter.

### 4.3. Statistical Analysis

Descriptive statistics were presented as absolute and relative frequencies for qualitative variables and as means with standard deviations (SD) and medians with ranges (minimum–maximum) for quantitative variables. The comparison of the quantitative variables in relation to the group variable was carried out using the non-parametric Mann–Whitney U test. Bivariate association between the quantitative variables were evaluated using Fisher’ exact test or Pearson’s chi-squared test. The primary outcomes were time to death and time to progression, defined as time from the date of diagnosis or diagnosis of disease progression or metastasis to the date of patient died and progressed. Loss of follow-up patients were considered as censored observation. Time-to-event data were estimated with the Kaplan–Meier estimator, and the Logrank test was applied to test for differences in survival distributions between groups. Univariable proportional hazards Cox models were fitted as a function of the binary PGCCs for each clinical outcome. For the analysis considering all patients, multivariable proportional hazards Cox models were fitted incorporating the PGCC variable along with known risk factors and confounders of the outcome (PGCC, disease, T, N, CTC/mL, PGCC/mL, and PGCC/CTC). Hazard ratios (HR) with 95% confidence intervals were estimated. Proportional hazards assumption was checked by using the scaled [29,30].

All hypothesis tests were two-sided at a 5% significance level. Thus, results with *p* values lower than 0.05 were considered statistically significant. No multiplicity adjustment was made for the outcomes since each constitutes a separate scientific question. All statistical analyses were conducted using statistical software R version 4.2 [31].

## Figures and Tables

**Table 1:** PGCC presence and clinical-pathological features.

	PGCC			
Clinical Features	Total *n* = 228	Negative *n* = 182	Positive *n* = 46	*p*
Gender				
Female	125 (100.00%)	96 (76.80%)	29 (23.20%)	0.237
Male	101 (100.00%)	84 (83.17%)	17 (16.83%)	
Missing	2	2	0	
Disease				
Metastatic	67 (100.00%)	47 (70.15%)	20 (29.85%)	**0.008**
Non-metastatic	152 (100.00%)	130 (85.53%)	22 (14.47%)	
Missing	9	5	4	
T grouped				
T1–T3	158 (100.00%)	131 (82.91%)	27 (17.09%)	0.471
T4	41 (100.00%)	32 (78.05%)	9 (21.95%)	
Missing	29	19	10	
N grouped				
N (−)	76 (100.00%)	64 (84.21%)	12 (15.79%)	0.524
N (+)	124 (100.00%)	100 (80.65%)	24 (19.35%)	
Missing	28	18	10	
CTC/mL				
Mean (SD)	4.03 (5.83)	3.72 (5.70)	**5.27 (6.22)**	**0.007**
Median (Range)	2.50 (0.00–51.00)	2.25 (0.00–51.00)	4.17 (0.16–39.66)	

**Table 2:** Univariable Cox regression analyses for Giant Cells and clinical-pathological features of all patients included in the analyses.

	Overall Survival			Progression-Free Survival		
Characteristic	nº Patients	HR * (95% CI)	*p*	nº Patients	HR * (95% CI)	*p*
PGCC	223			208		
No		— —			— —	
Yes		1.990 (1.087–3.644)	**0.023**		1.355 (0.796–2.309)	0.263
Disease	219			205		
Non-metastatic		— —			— —	
Metastatic		5.527 (3.120–9.792)	**<0.001**		5.567 (3.452–8.978)	**<0.001**
T grouped	199			186		
T1–T3		— —			— —	
T4		3.058 (1.598–5.852)	**0.001**		2.367 (1.378–4.066)	**0.002**
N grouped	200			187		
N (−)		— —			— —	
N (+)		5.521 (1.197–5.308)	**0.015**		3.760 (1.847–7.655)	**<0.001**
Gender	221			206		
Female		— —			— —	
Male		1.425 (0.828–2.451)	0.201		1.248 (0.791–1.970)	0.342
CTC/mL	223	1.013 (0.962–1.068)	0.621	208	1.011 (0.966–1.059)	0.642
PGCC/mL	223	1.341 (0.790–2.276)	0.277	208	1.371 (0.907–2.073)	0.134
PGCC/CTC	223	1.003 (0.968–1.441)	0.987	208	0.994 (0.748–1.322)	0.968

* HR = hazard ratio, CI = confidence interval.

**Table 3:** PGCC and CTC presence in all patients studied classified by tumor type.

Tumor Type		PGCC Presence		PGCC/mL		CTC Presence		CTC/mL		PGCC/CTC	
Epithelial	*N*	No	Yes	Mean (SD)	Range	No	Yes	Mean (SD)	Range	Mean (SD)	Range
Tumor											
Anal Canal	15	9	6	0.44 (0.82)	0.00–3.00	0	15	1.10 (1.11)	0.33–3.66	0.94 (2.35)	0.00–9.09
Colon	76	67	9	0.08 (0.29)	0.00–2.25	8	68	3.84 (4.09)	0.00–24.25	0.03 (0.13)	0.00–0.97
NSCLC	45	29	16	0.24 (0.52)	0.00–2.67	7	38	3.20 (3.42)	0.00–11.33	0.07 (0.17)	0.00–1.01
Gastric	51	42	9	0.11 (0.27)	0.00–1.00	3	48	5.45 (9.14)	0.00–51.00	0.10 (0.59)	0.00–4.17
Breast	29	25	4	0.06 (0.17)	0.00–0.67	7	22	4.74 (6.97)	0.00–30.50	0.03 (0.12)	0.00–0.67
Kidney	12	10	2	0.21 (0.58)	0.00–2.00	0	12	2.46 (2.43)	0.25–7.75	0.26 (0.63)	0.00–2.00
Total	228										

**Table 4:** Disease characteristics by type of carcinoma included.

Characteristics	Category	Anal Canal n (%)	Colon n (%)	NSCLC n (%)	Gastric n (%)	Breast n (%)	Kidney n (%)	Total n (%)
		15 (100.0)	76 (100.0)	45 (100.0)	51 (100.0)	29 (100.0)	12 (100.0)	228 (100.0)
**PGCC presence**	**No**	9 (60.00)	67 (88.16)	29 (64.44)	42 (82.35)	25 (86.21)	10 (83.33)	182 (79.82)
	**Yes**	6 (40.00)	9 (11.84)	16 (35.56)	9 (17.65)	4 (13.79)	2 (16.67)	46 (20.18)
**Metastatic**	**No**	11 (73.33)	71 (93.42)	7 (15.91)	34 (72.34)	23 (92.00)	6 (50.00)	152 (66.67)
	**Yes**	4 (26.67)	5 (6.58)	37 (84.09)	13 (27.66)	2 (8.00)	6 (50.00)	67 (29.38)
	**Missing**	0 (0.00)	0 (0.00)	1 (2.22)	4 (7.84)	4 (13.79)	0 (0.00)	9 (3.95)
**T**	**T1/T2**	5 (33.33)	28 (36.84)	19 (57.58)	9 (17.65)	16 (69.57)	6 (50.00)	83 (36.40)
	**T3/T4**	10 (66.67)	48 (63.16)	14 (31.11)	31 (60.78)	7 (30.43)	6 (50.00)	116 (50.88)
	**Missing**	0 (0.00)	0 (0.00)	12 (26.67)	11 (21.57)	6 (20.69)	0 (0.00)	29 (12.72)
**N**	**Negative**	5 (33.33)	39 (51.32)	6 (13.34)	8 (15.69)	10 (34.48)	8 (66.67)	76 (33.33)
	**Positive**	10 (66.67)	37 (48.68)	28 (62.22)	32 (62.74)	13 (44.83)	4 (33.33)	124 (54.39)
	**Missing**	0 (0.00)	0 (0.00)	11 (24.44)	11 (21.57)	6 (20.69)	0 (0.00)	28 (12.28)

Abbreviations. NSCLC = non-small cell lung cancer.

## Data Availability

Data is contained within the article and Supplementary Materials.

## Supplementary Materials

The following supporting information can be downloaded at https://www.mdpi.com/article/10.3390/ijms25189841/s1.
