# Supplementary material for: Circulating Polyploid Giant Cancer Cells, a Potential Prognostic Marker in Patients with Carcinoma
Source: Int J Mol Sci. 2024 Sep 11;25(18):9841. doi: 10.3390/ijms25189841 (PMC11432346; doi:10.3390/ijms25189841)
Supplement: Supplementary file 1 [file ijms-25-09841-s001.zip › ijms-3140672-supplementary.pdf]

Clinico-pathological features and follow up of all patients studied

Our data show that, as expected, metastatic disease, N+ tumors and T4 tumors are significantly associated with poor OS (HR=5.527, 95%CI: 3.120 to 9.792; HR=5.521, 95%CI: 1.197 to 5.308; HR=3.058 95%CI: 1.598 to 5.852, respectively), as well as with poor Progression Free Survival (PFS) (HR=5.567, 95%CI: 3.452 to 8.978; HR=3.760, 95%CI: 1.847 to 7.655; HR=2.367 95%CI: 1.378 to 4.066, respectively (**Table 2).**

As compared with patients without metastases, those with metastatic disease showed poorer OS (p<0.0001) **(Figure S1)** and poorer PFS (p<0.0001). 5-year PFS was 20% in patients with metastatic disease (95% CI: 9.4% to 41%) versus 74% (95% CI: 67% to 73%) in patients with non-metastatic disease (**Figure S2).**

According to multivariable Cox regression analysis, only metastatic disease was statistically significantly associated with poor OS (HR=3.39, 95%CI: 1.74 to 6.59, p<0001) and PFS (HR=3.64, 95%CI: 2.14 to 6.20, p< 0001).

Patients with tumors in stage T4 were found with poorer OS in comparison with patients with other stages (T1-T3) (p<0.001). In fact, 5-year OS was of 84% in patients with stage T1-T3 tumors (95% CI: 77% to 91%) versus 51% in patients with stage T4 tumors (95% CI: 35% to 74%). (**Figure S3).** A poorer PFS was found in patients with T4 tumors as compared with patients with other stages (T1-T3) (p=0.0013). 5-year PFS was 69% (95% CI: 62% to 78%) in patients with T1-T3 tumors versus 43% in patients with T4 tumors (95% CI: 28% to 65%) (**Figure S4).**

Patients with N positive tumors were found with poorer OS as compared with patients with N negative tumors (p=0.012). In fact, 5-year OS was 89% in patients with for N negative tumors (95% CI: 81% to 96%) versus 68% in patients with N positive tumors (95% CI: 58% to 79%) (**Figure S5).** Patients with N positive tumors showed poorer PFS as compared with patients with N negative tumors (p<0.0001). In fact, we observed a 5-year PFS of 85% in patients with N negative tumors (95% CI: 76% to 95%) versus 52% in patients with N positive tumors (95% CI: 43% to 63%) (**Figure S6).**

In patients with colon cancer, we observed a poorer OS in patients with T4 tumors as compared with patients with other stages (T1-T3) (p=0.045). In fact, the 5-year OS was 96% in patients with T1-T3 tumors (95% CI: 91% to 100%) versus 76% in patients with T4 tumors (95% CI: 52% to 100%) (**Figure S7).** We also observed poorer PFS in patients with stage T4 colon

cancer (5-year OS of 67%; 95% CI: 42% to 100%) as compared to patients with other stages (T1-T3 tumors; 85%, 95% CI: 76% to 94%; p=0.02).

Patients with N positive tumors showed poorer PFS (66%, 95% CI: 52% to 85%) as compared patients with N negative tumors (5-y OS of 97%, 95% CI: 91% to 100%), p<0.001 (**Figure S8).**

Patients with metastatic colon cancer showed, at 5 years, a statistically significant poorer PFS (p=0.008). In fact, 5 years PFS was 40% in patients with metastatic disease (95% CI: 14% to 100%) versus 86% (95% CI: 77% to 95%) in patients with non-metastatic disease (**Figure S9).**

Patients with metastatic gastric cancer showed a poorer OS versus non metastatic patients (p< 0.0001)**.** Patients with T4 gastric cancer also showed a poorer OS as compared with patients with other stages (T1-T3) (p= 0.014). In fact, 5-year OS was 60% for T1-T3 tumors (95% CI: 42% to 86%) versus 18% for T4 tumors (95% CI: 5.4% to 61%). We observed a 5-years PFS in patients with T4 tumors of 21% (95% CI: 7.1% to 62%) as compared to 57% in patients with T1-T3 tumors (95% CI: 39% to 83%) (p=0.017). However, patients with metastatic gastric cancer showed poorer PFS versus non metastatic patients (p<0.0001). Patients with metastatic gastric cancer showed a 5 years PFS of 21% (95% CI: 6.4% to 72%) versus 76% (95% CI: 62% to 92%) in patients with non-metastatic disease.

Patients with metastatic kidney cancer showed poor OS (p=0.022).

Supplementary Figures

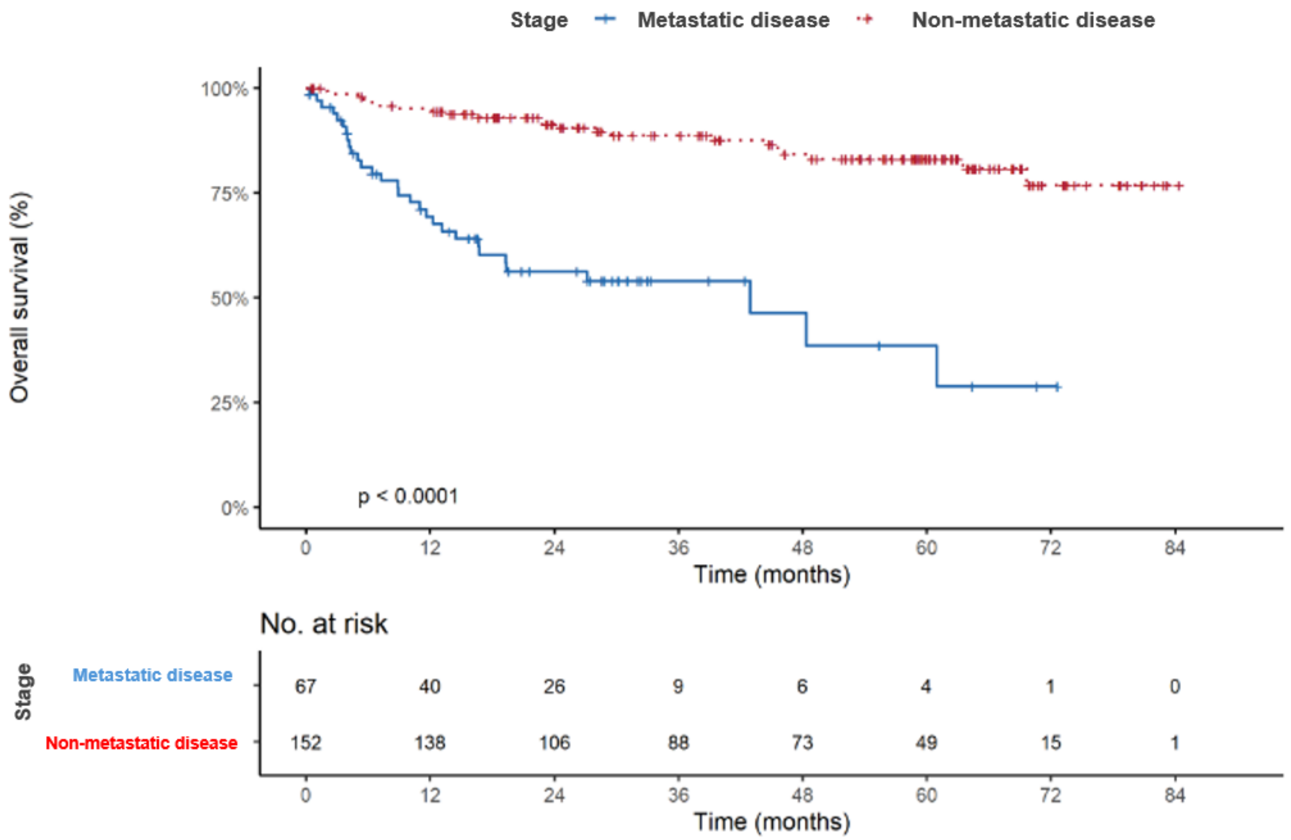

Supplementary Figure S1. Overall survival. Association between metastatic disease and poor OS in the whole group of patients.

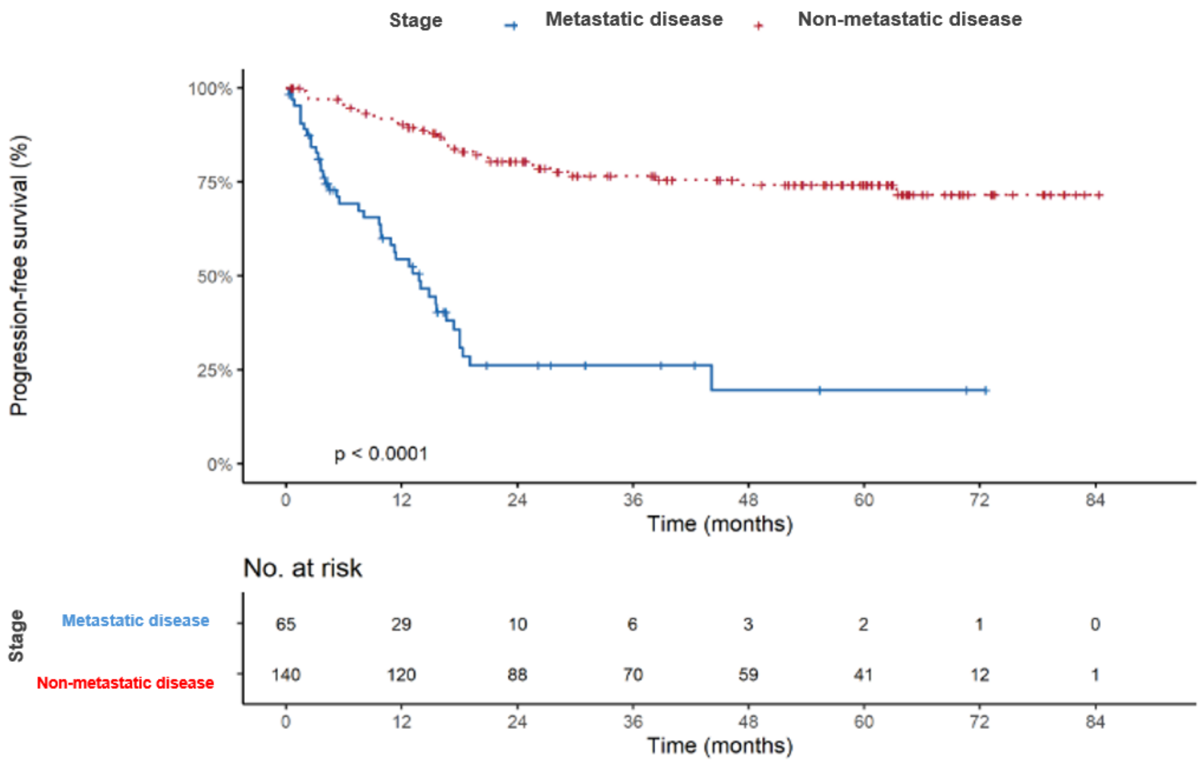

PFS rates (95%CI)							
**Characteristic**	**12 Month**	**24 Month**	**36 Month**	**48 Month**	**60 Month**	**72 Month**	**84 Month**
Stage							
Metastatic disease	54% (43% to 69%)	26% (16% to 42%)	26% (16% to 42%)	20% (9.4% to 41%)	20% (9.4% to 41%)	20% (9.4% to 41%)	— (— to —)
Non-metastatic disease	90% (85% to 95%)	81% (74% to 88%)	77% (69% to 85%)	74% (67% to 83%)	74% (67% to 83%)	72% (63% to 81%)	72% (63% to 81%)

Supplementary Figure S2. Progression-free survival. Association between metastatic disease and poor PFS in the whole group of patients.

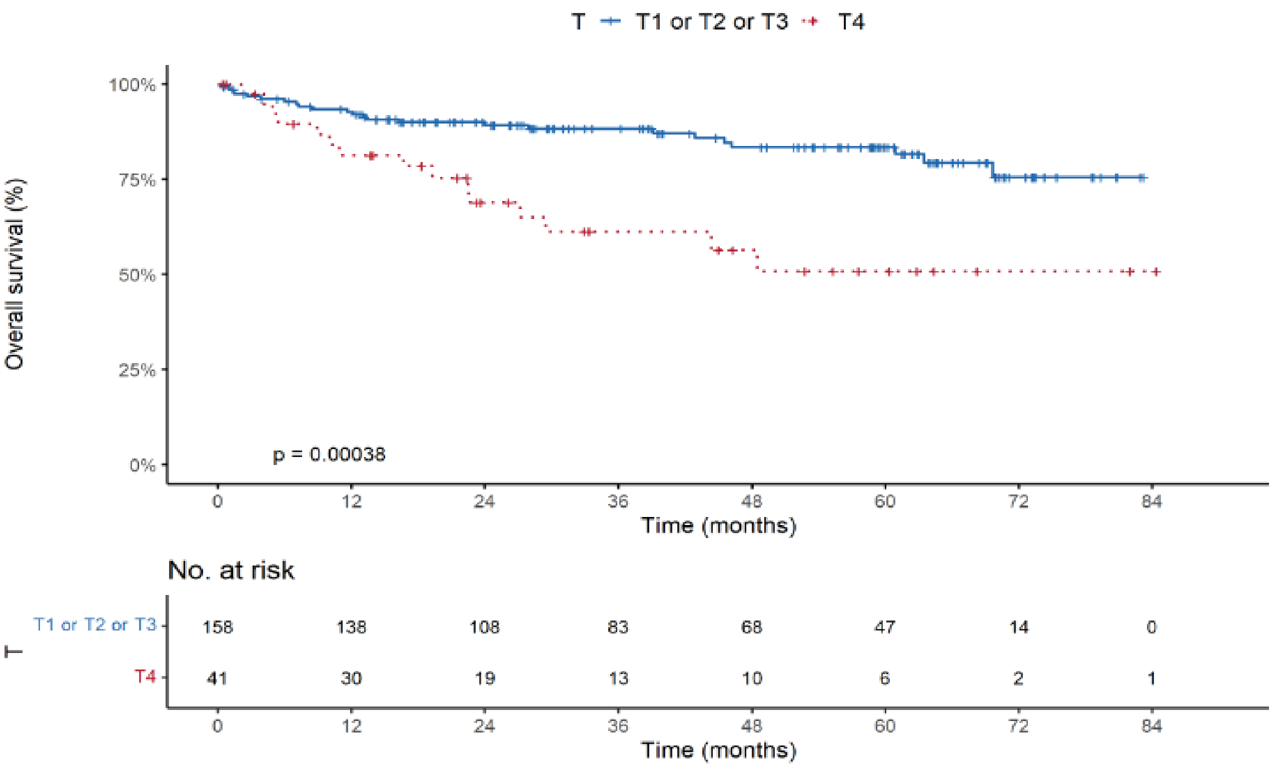

Survival rates (95%CI)							
**Characteristic**	**12 Month**	**24 Month**	**36 Month**	**48 Month**	**60 Month**	**72 Month**	**84 Month**
T4 tumors							
No	93% (89% to 97%)	89% (84% to 94%)	88% (83% to 94%)	84% (77% to 91%)	84% (77% to 91%)	75% (65% to 87%)	— (— to —)
Yes	81% (70% to 95%)	69% (55% to 86%)	61% (46% to 81%)	57% (41% to 78%)	51% (35% to 74%)	51% (35% to 74%)	51% (35% to 74%)

Supplementary Figure S3. Overall survival. Association between T4 tumors and poor OS in whole group of patients.

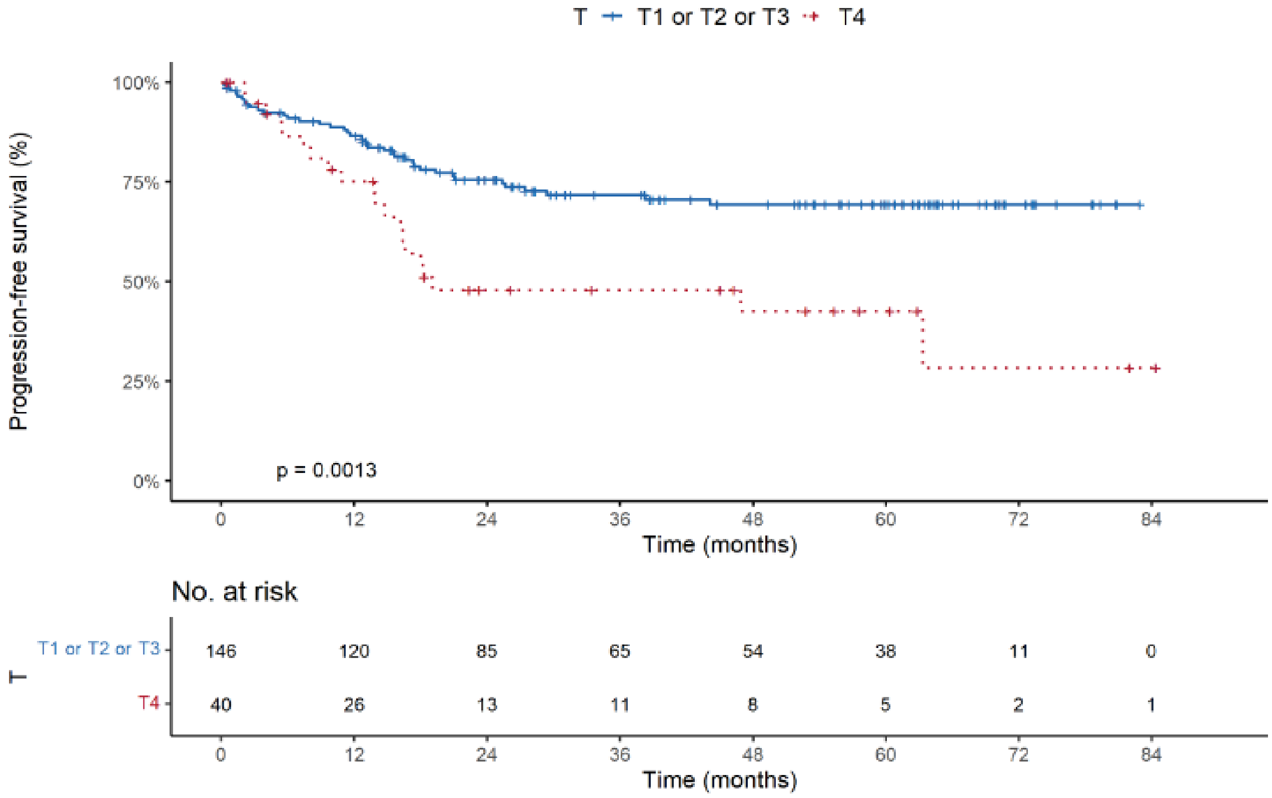

PFS rates (95%CI)							
**Characteristic**	**12 Month**	**24 Month**	**36 Month**	**48 Month**	**60 Month**	**72 Month**	**84 Month**
T4 tumors							
No	87% (81% to 92%)	76% (69% to 83%)	72% (64% to 80%)	69% (62% to 78%)	69% (62% to 78%)	69% (62% to 78%)	— (— to —)
Yes	75% (62% to 91%)	48% (34% to 68%)	48% (34% to 68%)	43% (28% to 65%)	43% (28% to 65%)	28% (12% to 70%)	28% (12% to 70%)

Supplementary Figure S4. Progression-free survival. Association between T4 tumors and poor PFS in whole group of patients.

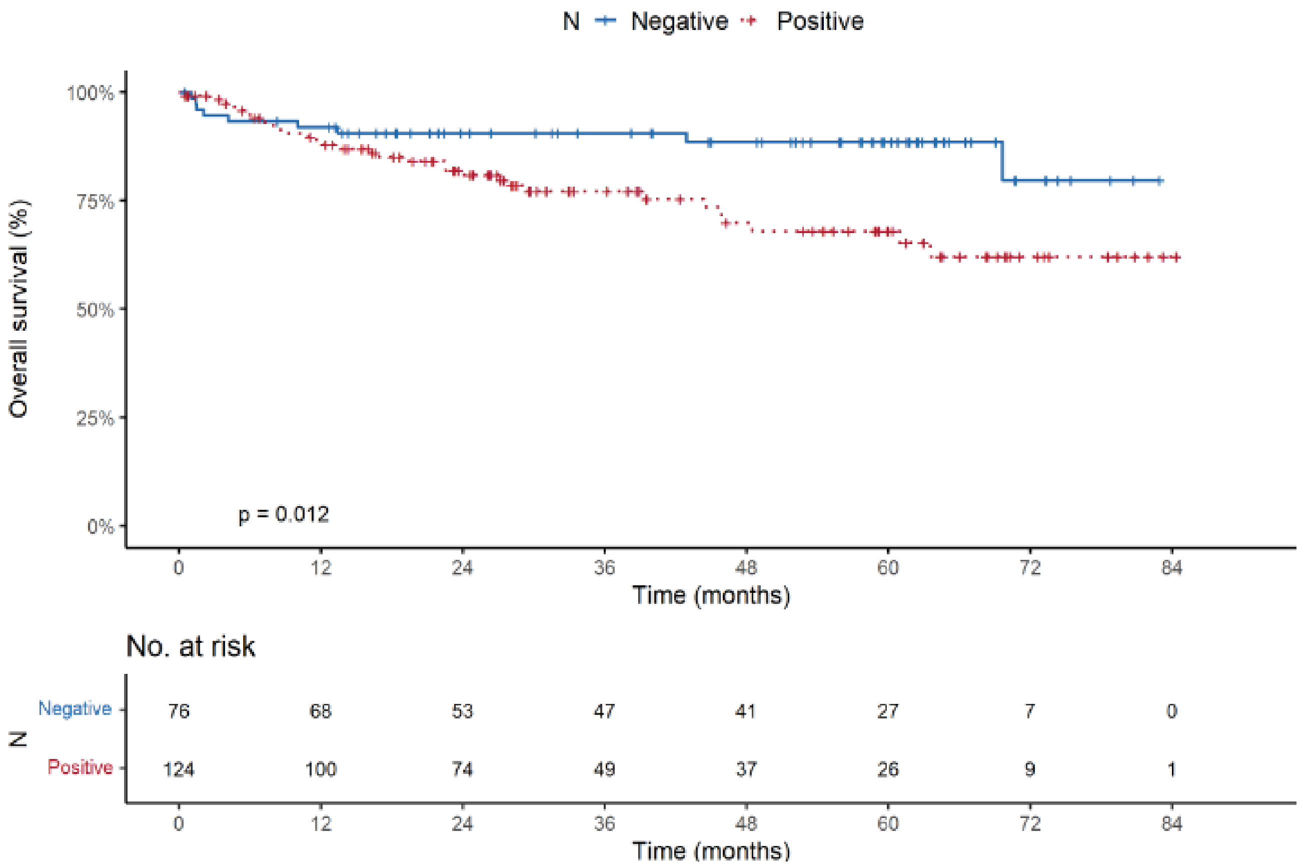

Survival rates (95%CI)							
**Characteristic**	**12 Month**	**24 Month**	**36 Month**	**48 Month**	**60 Month**	**72 Month**	**84 Month**
N grouped							
Negative	92% (86% to 98%)	91% (84% to 97%)	91% (84% to 97%)	89% (81% to 96%)	89% (81% to 96%)	80% (64% to 100%)	— (— to —)
Positive	89% (83% to 95%)	81% (74% to 89%)	77% (69% to 86%)	70% (61% to 81%)	68% (58% to 79%)	62% (51% to 76%)	62% (51% to 76%)

Supplementary Figure S5. Overall survival. Association between tumors N positives and poor OS in whole group of patients.

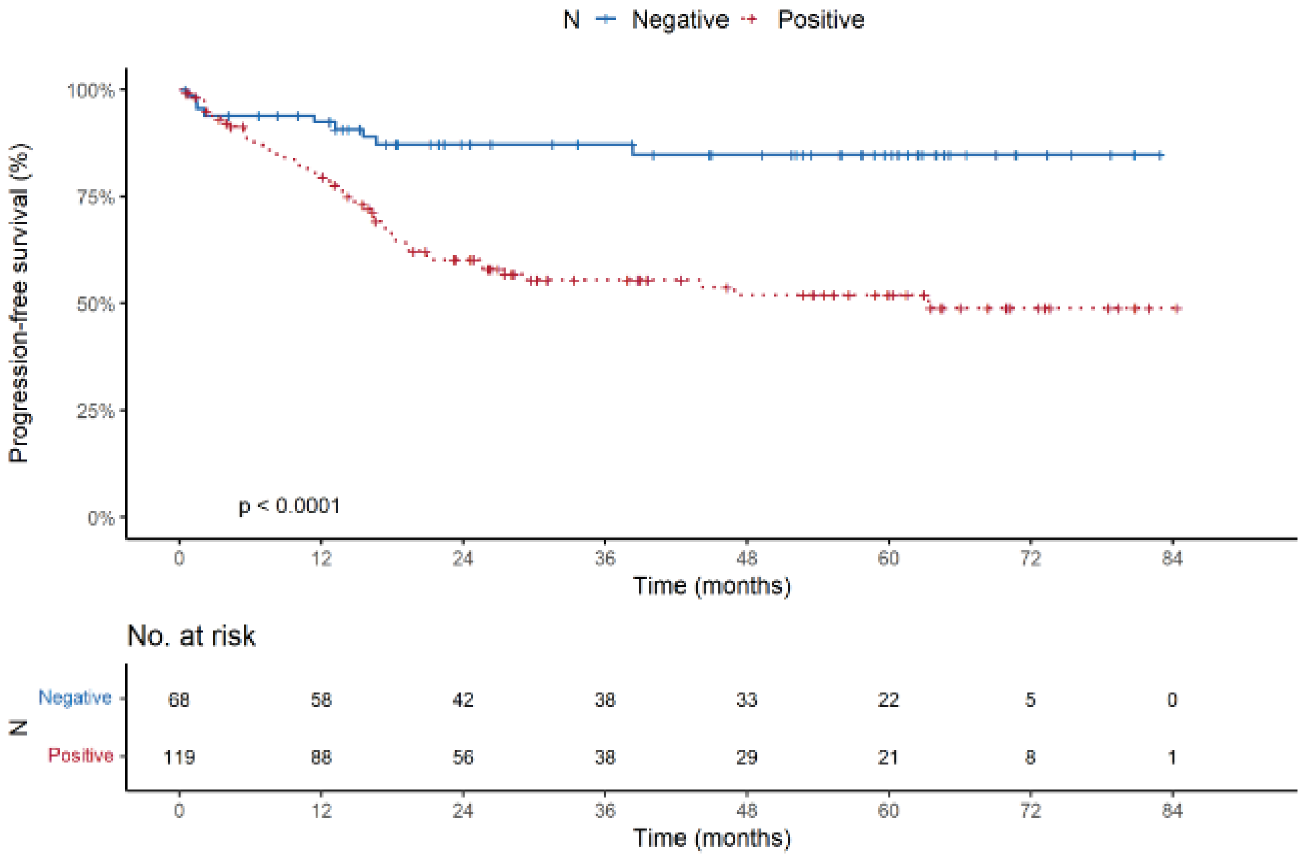

PFS rates (95%CI)

Characteristic	12 Month	24 Month	36 Month	48 Month	60 Month	72 Month	84 Month
N grouped							
Negative	92% (86% to 99%)	87% (79% to 96%)	87% (79% to 96%)	85% (76% to 95%)	85% (76% to 95%)	85% (76% to 95%)	— (— to —)
Positive	80% (72% to 87%)	60% (52% to 70%)	56% (47% to 66%)	52% (43% to 63%)	52% (43% to 63%)	49% (39% to 62%)	49% (39% to 62%)

Supplementary Figure S6. Overall survival. Association between tumors N positives and poor OS in whole group of patients.

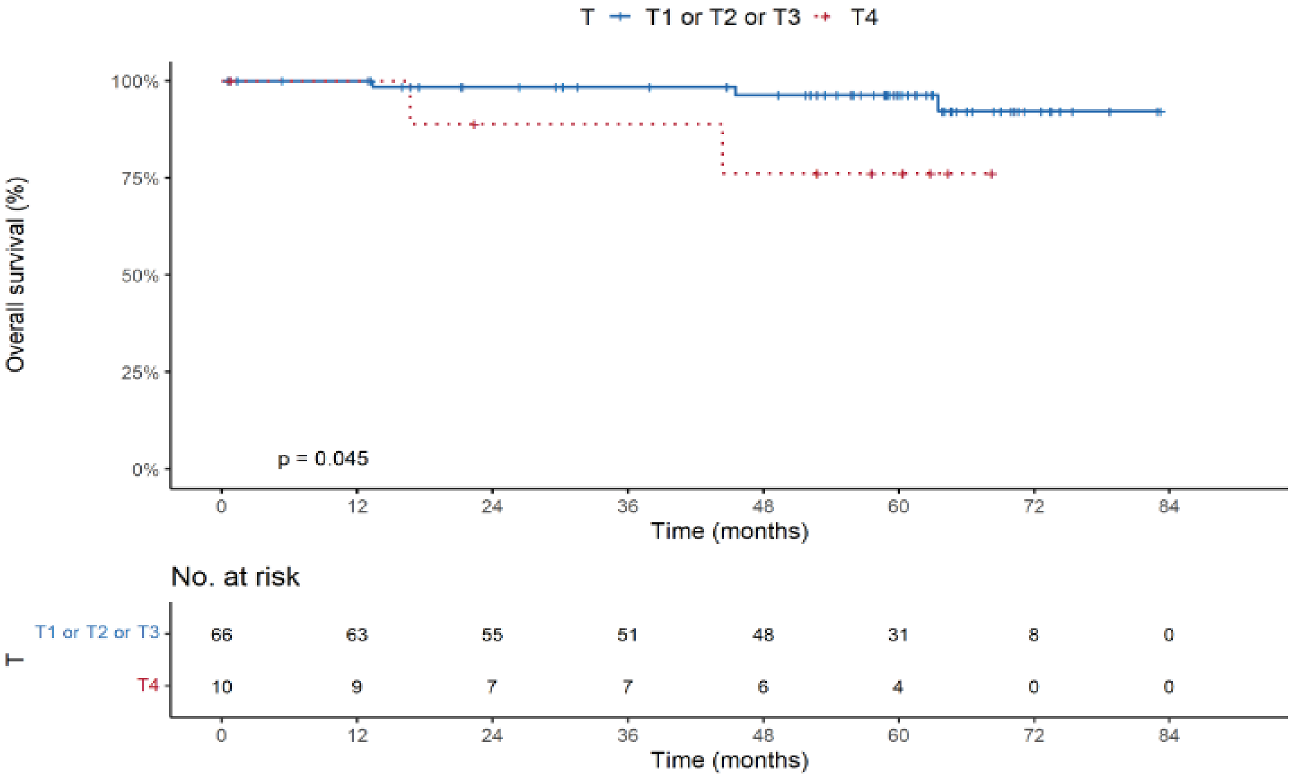

Survival rates (95%CI)							
**Characteristic**	**12 Month**	**24 Month**	**36 Month**	**48 Month**	**60 Month**	**72 Month**	**84 Month**
T4 tumors							
No	100% (100% to 100%)	98% (95% to 100%)	98% (95% to 100%)	96% (91% to 100%)	96% (91% to 100%)	92% (83% to 100%)	— (— to —)
Yes	100% (100% to 100%)	89% (71% to 100%)	89% (71% to 100%)	76% (52% to 100%)	76% (52% to 100%)	— (— to —)	— (— to —)

Supplementary Figure S7. Overall survival. Association between T4 tumors and poor OS in patients with colon cancer.

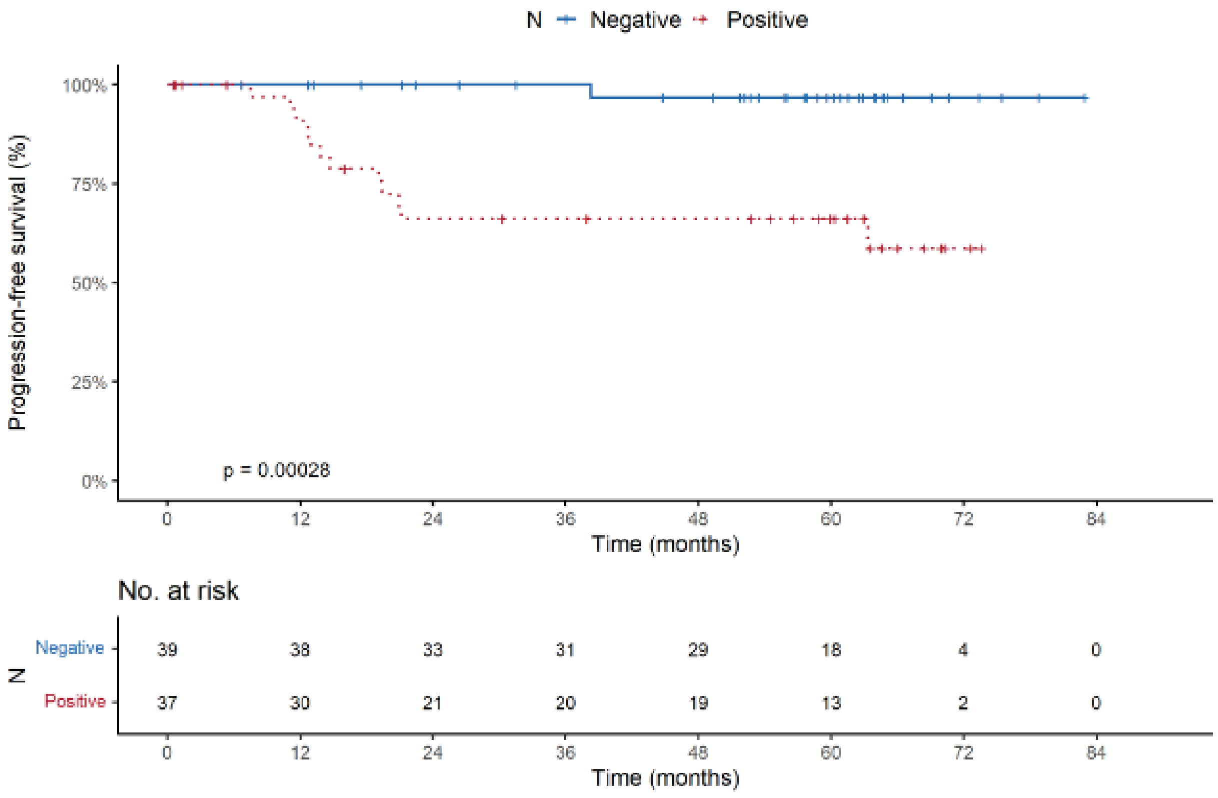

PFS rates (95%CI)							
**Characteristic**	**12 Month**	**24 Month**	**36 Month**	**48 Month**	**60 Month**	**72 Month**	**84 Month**
N grouped							
Negative	100% (100% to 100%)	100% (100% to 100%)	100% (100% to 100%)	97% (91% to 100%)	97% (91% to 100%)	97% (91% to 100%)	— (— to —)
Positive	91% (82% to 100%)	66% (52% to 85%)	66% (52% to 85%)	66% (52% to 85%)	66% (52% to 85%)	59% (42% to 82%)	— (— to —)

Supplementary Figure S8. Progression-free survival. Association between tumors N positives and poor PFS in whole group of patients.

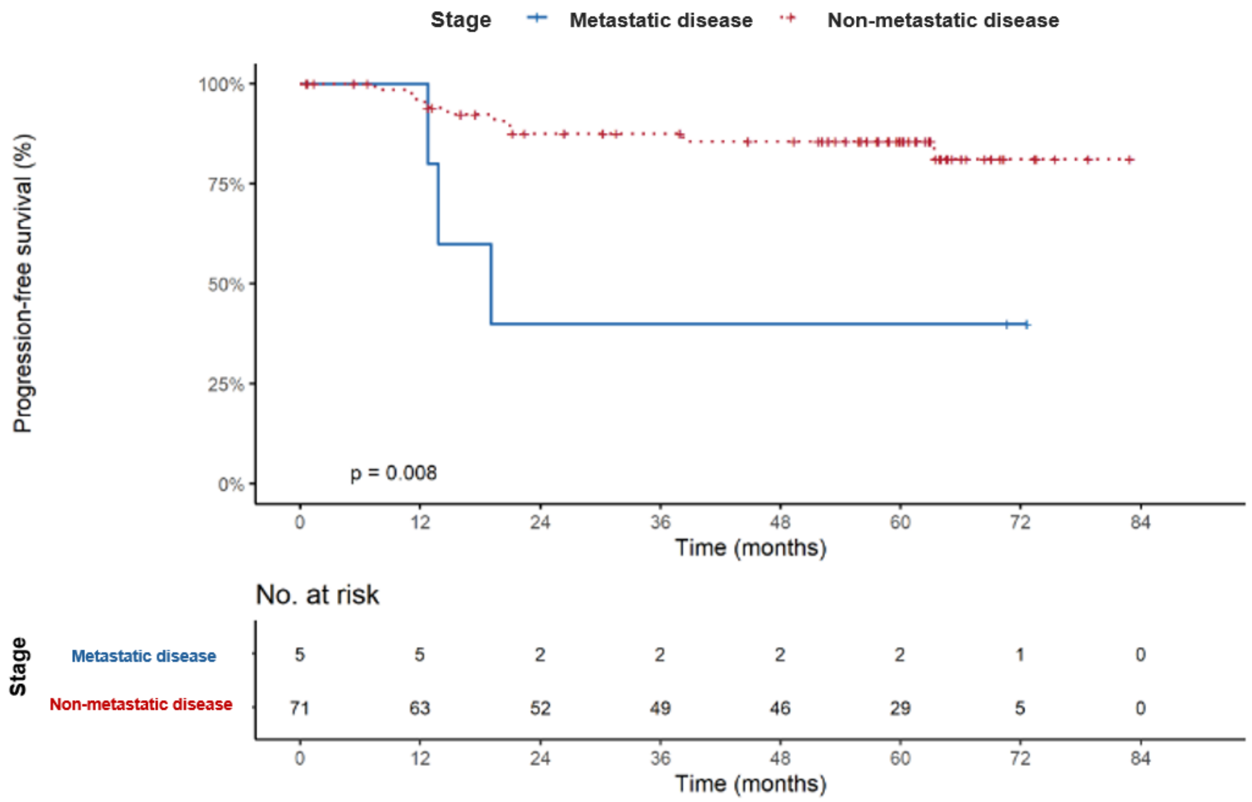

PFS rates (95%CI)							
**Characteristic**	**12 Month**	**24 Month**	**36 Month**	**48 Month**	**60 Month**	**72 Month**	**84 Month**
Stage							
Metastatic disease	100% (100% to 100%)	40% (14% to 100%)	40% (14% to 100%)	40% (14% to 100%)	40% (14% to 100%)	40% (14% to 100%)	— (— to —)
Non-metastatic disease	95% (91% to 100%)	88% (80% to 96%)	88% (80% to 96%)	86% (77% to 95%)	86% (77% to 95%)	81% (70% to 94%)	— (— to —)

Supplementary Figure S9. Progression-free survival. Association between metastatic disease and poor PFS in patients with colon cancer.
